# Antistaphylococcal Activity of the FtsZ Inhibitor C109

**DOI:** 10.3390/pathogens10070886

**Published:** 2021-07-13

**Authors:** Gabriele Trespidi, Viola Camilla Scoffone, Giulia Barbieri, Federica Marchesini, Aseel Abualsha’ar, Tom Coenye, Francesca Ungaro, Vadim Makarov, Roberta Migliavacca, Edda De Rossi, Silvia Buroni

**Affiliations:** 1 Department of Biology and Biotechnology “Lazzaro Spallanzani”, University of Pavia, 27100 Pavia, Italy; gabriele.trespidi01@universitadipavia.it (G.T.); viola.scoffone@unipv.it (V.C.S.); giulia.barbieri@unipv.it (G.B.); edda.derossi@unipv.it (E.D.R.); 2 Unit of Microbiology and Clinical Microbiology, Department of Clinical-Surgical, Diagnostic and Pediatric Sciences, University of Pavia, 27100 Pavia, Italy; federica.marchesini01@universitadipavia.it (F.M.); aseel.abu-alshaar01@universitadipavia.it (A.A.); roberta.migliavacca@unipv.it (R.M.); 3 Laboratory of Pharmaceutical Microbiology, Department of Pharmaceutical Analysis, Gent University, B-9000 Gent, Belgium; Tom.Coenye@ugent.be; 4 Department of Pharmacy, University of Naples Federico II, 80131 Naples, Italy; ungaro@unina.it; 5 Research Center of Biotechnology RAS, 119071 Moscow, Russia; makarov@inbi.ras.ru

**Keywords:** *Staphylococcus aureus*, drug resistance, biofilm, FtsZ

## Abstract

*Staphylococcus aureus* infections represent a great concern due to their versatility and involvement in different types of diseases. The shortage of available clinical options, especially to treat multiresistant strains, makes the discovery of new effective compounds essential. Here we describe the activity of the previously described cell division inhibitor C109 against methicillin-sensitive and -resistant *S. aureus* strains. Antibiofilm activity was assessed using microtiter plates, confocal microscopy, and in an in vitro biofilm wound model. The ability of C109 to block FtsZ GTPase activity and polymerization was tested in vitro. Altogether, the results show that the FtsZ inhibitor C109 has activity against a wide range of *S. aureus* strains and support its use as an antistaphylococcal compound.

## 1. Introduction

*Staphylococcus aureus* is a major human pathogen, being responsible for a variety of infections ranging from mild skin and soft tissue infections to severe pneumonia, osteomyelitis, endocarditis, and sepsis. It is often, although not exclusively, a hospital-acquired bacterium, and it can colonize medical implants through the formation of persistent biofilms [1].

*S. aureus* is also the most common opportunistic pathogen found in the lung of children with cystic fibrosis (CF). CF is a rare multiorgan genetic disorder, affecting more than 70,000 people worldwide, that causes impairments in the innate defense mechanisms of the lung and promotes the establishment of aggressive respiratory infections. *S. aureus* is often acquired by CF children within the first years after birth, and its presence has been associated with the development of early-stage lung disease. In particular, *S. aureus* infections induce an abnormal inflammatory response, further increased in case of coinfection with *Pseudomonas aeruginosa* and *Aspergillus* spp. [2], which causes an early lung function decline in infants [3]. Since the prevalence of *S. aureus* infections reaches 80% in children in the United States [4], and its chronic carriage in Europe ranges from 20 to 90%, excluding the United Kingdom where the antistaphylococcal antibiotic prophylaxis maintains the incidence below 10% [5], the correct management of these infections is of paramount importance.

To persist in the highly selective CF lung environment, this bacterium takes advantage of different adaptive strategies, such as in the case of hypermutator strains [6], the intracellular persistence [7,8], the switch to auxotrophic small colony variant phenotype [9], and the formation of biofilms. Biofilms are organized microbial communities embedded in a self-produced hydrated matrix of polymeric molecules, mostly polysaccharides, proteins, and DNA. Within this complex structure, bacteria become highly tolerant to the host’s immune response, antibiotic treatments, and several other environmental stresses [10]. In *S. aureus*, the main component of the matrix is the poly-N-acetyl-1,6 glucosamine polysaccharide (PNAG), even though extracellular DNA, teichoic acids, and several surface proteins are present [10]. During CF lung infections, this bacterium aggregates in multicellular clusters embedded in PNAG within the mucus [11]. Furthermore, an increased ability to form biofilm can be the result of convergent phenotypic evolution of different *S. aureus* isolates from chronic CF infections, underlying the importance of this trait for long-term persistence [8].

The already challenging management of these infections is worsened by the constantly increasing isolation of methicillin-resistant *S. aureus* (MRSA) strains [4]. First isolated in 1961, MRSA has recently also emerged as a threat for CF patients since it has a significantly higher clinical impact compared to its methicillin-sensitive counterpart. Indeed, MRSA respiratory infections are associated with increased hospitalization and higher risk of death [12,13]. The limited therapeutic options available for their eradication require the extensive use of trimethoprim–sulfamethoxazole, which can induce the formation of highly resistant thymidine-dependent small colony variants [9], and vancomycin [14]. Unfortunately, it is demonstrated that the prolonged use of this glycopeptide in these patients leads to the appearance of heteroresistant vancomycin-intermediate *S. aureus* (hVISA) strains, making their eradication virtually impossible [15].

The increasing incidence of resistant *S. aureus* strains is a major global issue, and it drastically reduces the effectiveness of the current antibiotic therapies against several non-CF human infections as well. Among those, chronic wound infections are a neglected healthcare problem that is actually becoming a significant socioeconomic burden. Indeed, the worldwide increase of obesity means a similar rise in the prevalence of diabetes and cardiovascular diseases, which are associated with the development of lower extremity nonhealing wounds, such as chronic venous, diabetic, and pressure ulcers [16]. Therefore, it has been estimated that around 1–2% of the population of developed countries suffers from these conditions [17]. Chronic wounds are normal wounds arrested in the inflammation stage of the healing process, where the prolonged presence of activated polymorphonuclear leukocytes causes extensive damage to the host tissue [18]. In particular, *S. aureus* is the most common microorganism found in the chronic wound microenvironment, colonizing up to 90% of leg and diabetic foot ulcers [19,20]. Moreover, the presence of MRSA biofilms is associated with enhanced virulence, antibiotic therapy failure, and worse overall prognosis [21,22].

A few years ago, we characterized a newly synthesized benzothiadiazole molecule, C109, as a promising antimicrobial active against the extremely drug-resistant Gram-negative *Burkholderia cenocepacia*, a notorious CF pathogen [23]. Afterward, C109 was found to be a broad-spectrum bactericidal compound, active against several Gram-positive and -negative pathogens, including MRSA [24]. Being considered a high-potential antimicrobial, an inhalable nanoformulation, with a great biofilm inhibition effect against *B. cenocepacia*, was developed for local treatment of CF lung infections [25]. Moreover, a deep characterization of C109 was performed, demonstrating that the benzothiadiazole molecular scaffold has a very good potential for the development of new, more potent antistaphylococcal compounds, since different chemically modified derivatives showed similar or even improved activity against *S. aureus* [26]. The great potentiality of this molecule is also given by its molecular target, the extremely conserved tubulin-like FtsZ protein [24]. C109 inhibits the GTPase activity of *B. cenocepacia* and *P. aeruginosa* FtsZ, consequently blocking the formation of the polymers [24,26]. The ubiquity and the essential function of FtsZ make it an attractive drug target, as attested by the increasing number of FtsZ inhibitors characterized within the last few years [27]. Furthermore, the design of novel antimicrobials may take advantage of the in-depth study of the FtsZ interactome [28]. Indeed, several division protein interactions are essential for bacterial survival, and their impairment is emerging as a novel approach for future drug development [29].

In this work, the antimicrobial potential of C109 against a large group of sensitive (methicillin-sensitive *Staphylococcus aureus* (MSSA)) and resistant (MRSA) clinical isolates was characterized, and the minimum inhibitory concentrations (MICs) and the minimum bactericidal concentrations (MBCs) of the compound were determined. Its biofilm inhibition and eradication activity towards *S. aureus* were assessed using 96-well microtiter plates. The C109 effect on the bacterial biofilm was further visualized by confocal microscopy, and three-dimensional high-resolution images were obtained. Then, the compound was tested against the *S. aureus* Mu50 strain in an in vitro biofilm wound model, and its biofilm inhibitory activity was assessed in this microenvironment. Finally, its ability to block FtsZ GTPase activity was tested in vitro. The inhibition of FtsZ monomer polymerization in the presence of different concentrations of C109 was also characterized using an in vitro sedimentation assay.

## 2. Results

### 2.1. Activity of C109 against Sensitive (MSSA) and Resistant (MRSA) S. aureus Strains

The activity of C109 was tested in quadruplicate by microdilution method in Mueller Hinton (MH) broth against a total of 102 *S. aureus* strains, 51 (50%) of which were MRSA (Tables S1 and S2, Supplementary Materials). The bacterial load in the 250 μL broth final volume was always between 1.5 × 10^4^ and 3.5 × 10^5^ CFU/mL. The minimal inhibitory concentration (MIC) of C109 against the reference strain *S. aureus* ATCC 25923 was 2–4 µg/mL, while the minimal bactericidal concentration (MBC), tested under the same conditions, was 4–16 µg/mL.

**Figure 1:**
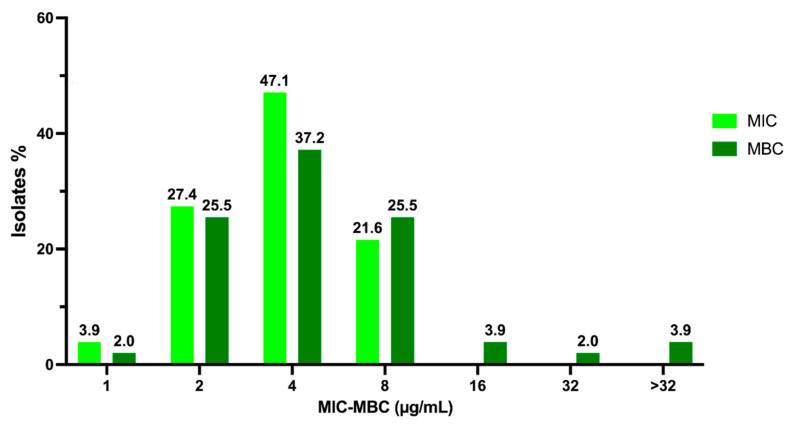
Overview of C109 MIC and MBC values obtained for MSSA strains.

Among the 51 MSSA strains, 2 (3.9%), 14 (27.4%), 24 (47.1%), and 11 (21.6%) showed MIC values of 1, 2, 4, and 8 µg/mL, respectively (Figure 1). These results show that the most representative MIC value among clinical isolates is the same as the reference strains, i.e., 4 µg/mL. The MBCs for the same MSSA strains were 1, 2, 4, 8, 16, 32, and >32 µg/mL for 1 (2%), 13 (25.5%), 19 (37.2%), 13 (25.5%), 2 (3.9%), 1 (2%), and 2 (3.9%) strains, respectively (Figure 1). Interestingly, these results demonstrate that 4 µg/mL is also the most represented value for the MBC.

**Figure 2:**
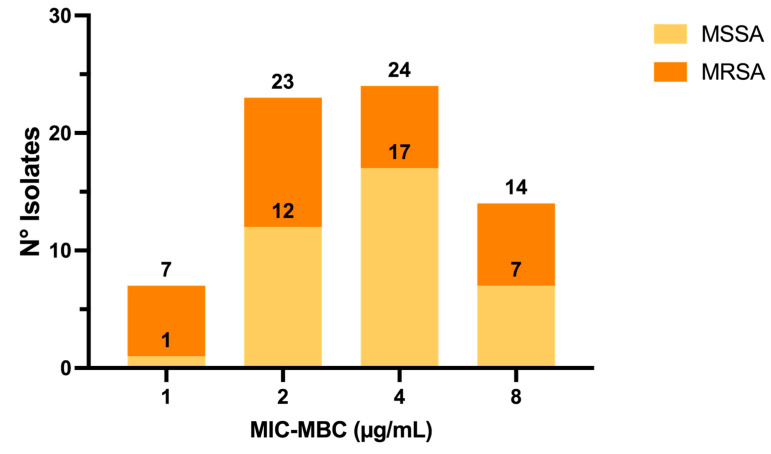
Number of *S. aureus* isolates for which the C109 MIC values caused bacterial death (MIC = MBC).

Indeed, for *n* = 37/51 (72.55%) MSSA, i.e., for most strains, the C109 MIC showed bactericidal activity (MIC = MBC), while the remaining 27.45% MSSA strains showed MBC values higher than the MICs (Figure 2).

**Figure 3:**
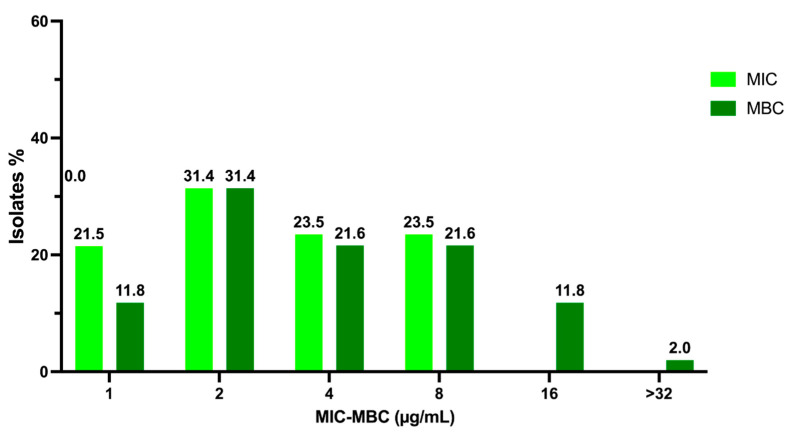
Overview of C109 MIC and MBC values obtained for MRSA strains.

Among the 51 MRSA isolates, 11 (21.5%), 16 (31.4%), 12 (23.5%), and 12 (23.5%) showed MIC values of 1, 2, 4, and 8 µg/mL, respectively (Figure 3). Interestingly, the most represented result is 2 µg/mL, i.e., below the one achieved for sensitive strains. The MBCs for these strains were 1, 2, 4, 8, 16, 32, and >32 µg/mL for 6 (11.8%), 16 (31.4%), 11 (21.6%), 11 (21.6%), 6 (11.8%), and 1 (2%), respectively (Figure 3). In this case, the most representative value is also 2 µg/mL.

For *n* = 31/51 (60.8%) MRSA strains, the C109 MIC showed bactericidal activity (MIC = MBC), while the remaining 20/51 (39.2%) MRSA strains showed MBC values higher than the MICs, similarly to the results achieved with MSSA strains (Figure 2).

### 2.2. C109 Biofilm Inhibitory Activity against S. aureus ATCC 25923

**Figure 4:**
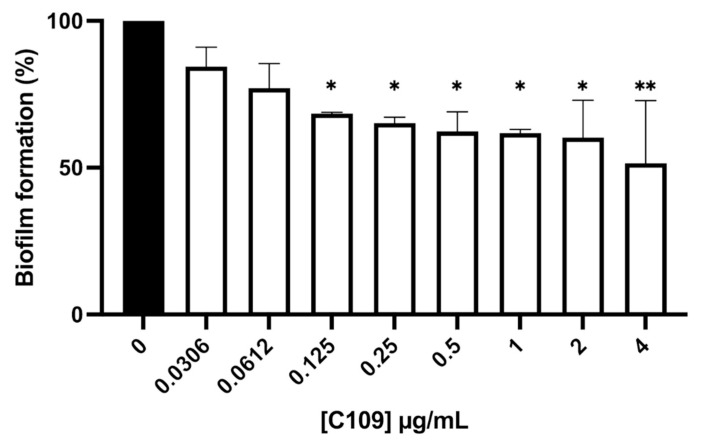
Effect of C109 against *S. aureus* ATCC 25923 biofilm formation. Bacterial biofilms were grown in the absence (black bar) or in the presence (white bars) of increasing concentrations of C109, indicated below each bar. The results are expressed as % of residual biofilm formation (mean ± standard error, *n* = 3). * *p* < 0.1; ** *p* < 0.01 (one-way ANOVA test).

In order to determine the biofilm inhibitory activity of the compound C109 against *S. aureus*, the 96-well microplate crystal violet staining assay was carried out. Increasing concentrations of C109 were tested, ranging from 0.0306 to 4 μg/mL. In these conditions, the treatment with the compound C109 induced a statistically significant decrease in the biofilm formation at 0.125 μg/mL (Figure 4).

To assess C109 biofilm eradication potential, mature *S. aureus* biofilms grown in 96-well microtiter plates were treated with 16 (4× MIC) and 32 (8× MIC) μg/mL of the compound for 24 h. Unfortunately, even using the highest concentration, C109 was not able to eradicate the mature *S. aureus* biofilm (Figure S1, Supplementary Materials).

**Figure 5:**
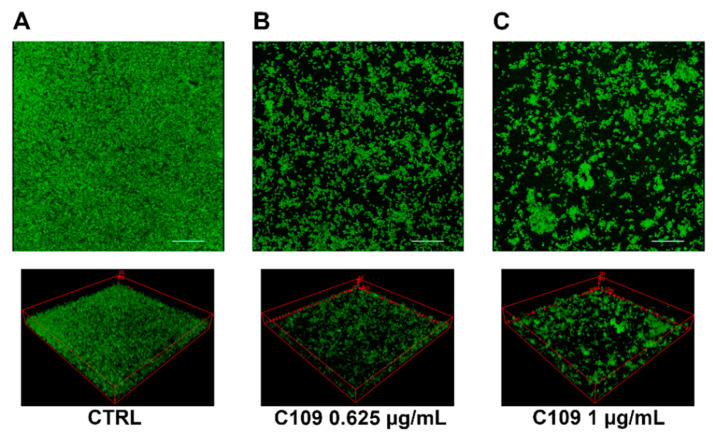
CLSM images of *S. aureus* ATCC 25923 biofilms grown in a Lab-Tek II Chamber Slide. Pictures were taken with an overall magnification of 400×. Cells were grown overnight at 37 °C in TSB + 1% glucose with no C109 (CTRL, (**A**)), 0.625 μg/mL of C109 (**B**), or 1 μg/mL of C109 (**C**). Eighty planes at equal distances along the Z-axis of the biofilm were imaged by CLSM. These 2D images were stacked to reconstruct the 3D biofilm image. Scale bar represents 28 μm.

To better characterize the effects of the compound C109 on *S. aureus* biofilm morphology, a confocal laser scanning microscopy (CLSM) analysis was performed. *S. aureus* ATCC 25923 biofilms were grown as static cultures in three parallel chambered coverglasses, in the absence or presence of different concentrations of C109 (0.625 and 1 μg/mL), at 37 °C. Biofilms were stained with the nucleic acid staining Syto9. A representative 3D image reconstruction of the biofilm from each C109 concentration is shown in Figure 5. This preliminary analysis highlighted that *S. aureus* biofilms formed in the absence of compound were able to almost completely cover the surface of the field (Figure 5A). On the contrary, significant morphological differences were observed in presence of both concentrations of C109: biofilms were less structured, contained fewer cells, and were unable to colonize the entire surface of the well (Figure 5B,C).

**Figure 6:**
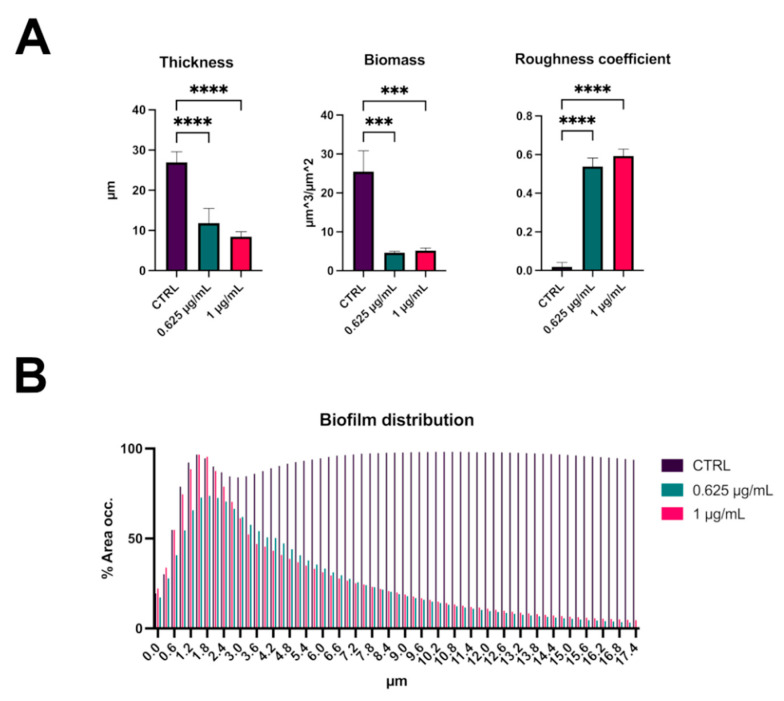
Analysis of biofilm properties by COMSTAT 2. Measures of average thickness, total biomass, and roughness coefficient (**A**); % of the area occupied by biofilm distribution (**B**). Data are the mean ± SD of the results from three independent replicates. *** *p* < 0.001, **** *p* < 0.0001 (one-way ANOVA test).

The analysis of biofilm properties was carried out with COMSTAT 2. This analysis revealed that *S. aureus* biofilm had a medium thickness of 25 μm, while the thickness and the biomass decreased significantly in presence of the compound C109 (Figure 6A). At the same time, the roughness coefficient (variation in thickness calculated from the distribution of the biofilm thickness), which provides an indicator of biofilm heterogeneity, increased significantly (Figure 6A). Moreover, the biofilm distribution on the Z-axis of the treated samples changed drastically, and it occupied only the first layer of the chamber (Figure 6B).

### 2.3. C109 Biofilm Inhibitory Activity against S. aureus Mu50

**Figure 7:**
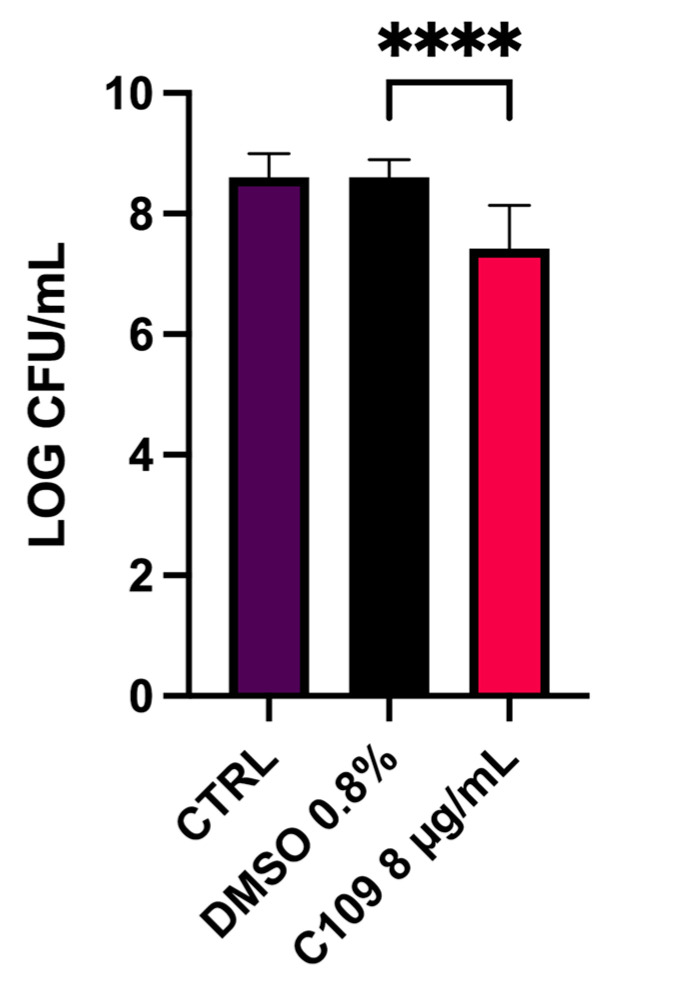
Effect of C109 against *S. aureus* Mu50 biofilm formation in a biofilm wound model. Bacterial biofilms were grown in the presence of 8 μg/mL of C109 or DMSO 0.8%. The results are expressed as log CFU/mL (mean ± standard error, *n* = 12) recovered after 24 h of incubation. **** *p* < 0.0001 (one-way ANOVA test).

Given the good biofilm inhibition achieved in the 96-well microplate experiment against the MSSA strain, the biofilm inhibitory activity of the compound C109 was further tested on the MRSA strain Mu50 in an in vitro biofilm wound model of chronic infection. This consists of an artificial dermis (AD) soaked in a medium containing blood and plasma and mimicking the nutritional condition of an infected wound [30]. In this model, the treatment with 8 μg/mL (2× MIC) after 24 h of incubation resulted in a statistically significant reduction in the CFU/mL (15 fold) compared to the untreated and the solvent-only controls (Figure 7). The same results were achieved using a C109 formulation consisting of nanocrystals stabilized with D-α-tocopheryl polyethylene glycol 1000 succinate embedded in hydroxypropyl-β-cyclodextrin [25] (data not shown).

### 2.4. C109 Activity on Purified S. aureus FtsZ

It has been previously demonstrated that C109 targets the FtsZ protein of *B. cenocepacia* and of *P. aeruginosa*, blocking their GTPase and polymerization activities [24,26]. To assess C109 activity against the purified FtsZ of *S. aureus*, the wild type and a previously described more stable deleted version of the protein (12-316aa, ΔFtsZ) [31] were expressed and purified, as described in Section 4.

**Figure 8:**
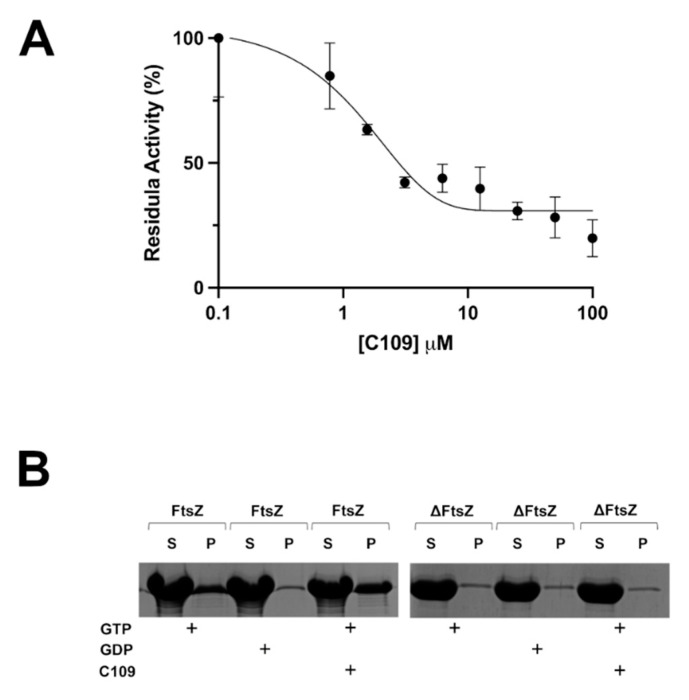
GTPase activity and polymerization assays demonstrate that C109 is an inhibitor of S. *aureus* FtsZ. (**A**) IC_50_ determination of C109 against wild-type FtsZ. (**B**) SDS-PAGE of the sedimentation assay of the wild-type FtsZ and of the ΔFtsZ in the presence (+) or absence of C109. P, unsoluble fraction (pellet); S, soluble fraction (supernatant). Data are the mean ± SD of the results from three different replicates; images are representative of at least three different experiments.

The GTPase activity of recombinant FtsZ of *S. aureus* was tested using a coupled spectrophotometric assay, as previously described [24]. The enzymatic assay revealed that C109 inhibits the wild-type FtsZ GTPase activity with a 50% inhibitory concentration (IC_50_) of 1.5 μM (Figure 8A), which indicates that the protein activity is impaired by C109. On the contrary, the IC_50_ of C109 against ΔFtsZ was not determinable (data not shown), showing a lack of C109 activity towards this version of the protein.

To understand whether the in vitro polymerization activity of *S. aureus* FtsZ was impaired by C109, a previously described sedimentation protocol was used [24]. As expected, the wild-type FtsZ of *S. aureus* polymerized very well in vitro in presence of GTP and not in presence of GDP. However, this activity was not blocked by the presence of C109, even at the highest concentration (Figure 8B). On the other hand, in the same conditions, the ΔFtsZ was not able to polymerize in the presence of its substrate GTP, neither in the presence nor in the absence of C109.

These results showed that the compound C109 interferes only with the GTPase activity of *S. aureus* FtsZ, contrary to what was previously described in *B. cenocepacia*, in which C109 blocks both activities of the protein [24]. Indeed, the percentage of identity of the two proteins is 46%, and their C-terminal domains, in particular, are very different (Figure S2, Supplementary Materials).

## 3. Discussion

*S. aureus* opportunistic infections are among the most common causes of morbidity and mortality worldwide. Indeed, this bacterium, usually found on the skin and in the nasal cavity as a human commensal, can cause diverse invasive infections, especially in hospitalized and immunocompromised patients. The alarming increase in MRSA strain isolation greatly limits the therapeutic options effective against these infections and leads to increased mortality [32]. Cystic fibrosis patients are among the most affected populations, being highly susceptible to *S. aureus* lung infections prevalently at a young age [4,5]. Moreover, this microorganism is often the most common colonizer of chronic wounds, playing a role in the healing delay [22].

To tackle the problem of the shortage of antibiotics active against *S. aureus* infections, in this study we demonstrated the promising antimicrobial activity of the compound C109, firstly against several MSSA and MRSA clinical isolates by MIC and MBC determination. The strains were obtained from different samples (wound, skin, vaginal, ulcer, ear swabs, urine, blood culture, sputum, bronchial aspirate) from two hospitals in Northern Italy (IRCCS Policlinico San Matteo, Pavia, and Alessandro Manzoni Hospital, Lecco). The MIC values for these strains ranged between 1 and 8 μg/mL, which are considered low concentrations, given the increasing isolation of multiresistant strains insensitive to most of the currently prescribed antistaphylococcal antibiotics [15,33]. Interestingly, most strains showed MIC = MBC, and the MIC and MBC values obtained for MRSA strains were lower than those obtained for MSSA strains, indicating the great potential of C109 for the treatment of drug-resistant bacteria. More importantly, C109 appears as one of the most effective new molecules published within the last few years against MSSA and MRSA strains, showing an antimicrobial effect considerably higher than some natural compounds [34,35] and antimicrobial peptides [36] and a lower cytotoxicity towards human cells [23,35].

Since *S. aureus* is widely known to form recalcitrant biofilms during chronic infections [10,37], C109 biofilm inhibitory activity was assessed. The compound was active at a concentration 128-fold lower than the MIC, and it greatly inhibited the formation of the bacterial biofilm in vitro at 0.125 μg/mL. These results showed increased activity of C109 in *S. aureus* biofilm inhibition compared to that previously reported against *B. cenocepacia* [24,25], confirming the great potentiality of the molecule against this microorganism [26]. Moreover, *S. aureus* biofilms were visualized by confocal laser scanning microscopy, highlighting significant differences in the biofilm structure of C109-treated cells compared to the untreated control. The biofilms were thinner and less structured in the presence of C109: indeed, the compound led to the formation of highly heterogeneous biofilms that were unable to cover the entire abiotic surface and contained fewer bacteria compared to the control. Since the presence of biofilm-growing bacteria is often associated with antibiotic treatment failure [38], our results point out the potential of C109 as a novel biofilm inhibitor, able to prevent *S. aureus* biofilm establishment and, consequently, increase the effectiveness of the canonical therapies.

Unfortunately, despite the promising biofilm inhibitory potential, C109 was not able to eradicate *S. aureus* mature biofilms in vitro, even at high concentrations, probably due to the poor penetration of the compound into the biofilm matrix. This corroborates the results obtained for *B. cenocepacia* [25] and confirms once more the extreme tolerance to antimicrobials acquired by bacteria growing in these organized communities, underlying the importance of prevention measures.

The uncontrolled spreading of MRSA strains in hospitals and communities is a serious healthcare issue that leads to an increased prevalence of dangerous multiresistant infections in the general population. Diabetic foot ulcer patients are particularly susceptible to these infections [20] that, once established, become extremely hard to eradicate due to the formation of persistent biofilms [22,37]. For this reason, the biofilm inhibitory potential of C109 was tested in a biofilm wound model against the Mu50 MRSA strain. In this way, it was possible to validate the effect of the compound in a model reflecting the nutritional condition in wounds, where the physiological environment might potentially interfere with the antimicrobial activity [39]. In this case, C109 was effective in the inhibition of the formation of biofilm by this multiresistant strain, leading to a substantial reduction in the biofilm-forming bacteria using a concentration 2-fold greater than the MIC. These data confirm its great in vitro potential against MRSA and pave the way to the development of a C109 formulation for topical application that virtually represents an alternative treatment for these difficult-to-treat infections.

To have a more complete overview of C109 antimicrobial activity, we also biochemically characterized the effect of the compound on its target at the molecular level. We previously identified the target of this broad-spectrum antibacterial in the Gram-negative rods *B. cenocepacia* and *P. aeruginosa* as the division protein FtsZ [24,26]. Indeed, C109 acts on the GTPase activity of the protein and prevents the polymerization [24]. The compound showed a potent GTPase inhibitory activity towards the full-length *S. aureus* FtsZ, showing a lower IC_50_ value (1.5 μM) compared to FtsZ of *B. cenocepacia* (IC_50_: 8.2 μM) and *P. aeruginosa* (IC_50_: 3.5 μM) [26]. Surprisingly, when repeating the same assay on the truncated *S. aureus* FtsZ, which did not possess the intrinsically disordered C-terminal tail (CTT), C109 was unable to block the enzymatic activity of the protein. Since both proteins had a comparable GTPase activity [31], these results suggest an involvement of the CTT in the C109 molecular interaction with its target. However, given the hypervariability of the C-terminal linker, the region of the CTT that links the FtsZ globular catalytic domain and the C-terminal peptide, among FtsZs from different species [40], it is unlikely that a broad-spectrum compound, such as C109, binds directly to this region. Moreover, none of the characterized FtsZ inhibitors act directly on this region [27], although the effect of some molecules, such as PC190723, can influence the CTT conformation, with dramatic effects on FtsZ function [41]. Thus, the CTT could have an indirect role in the C109 inhibition of the GTPase activity since it is reported that the FtsZ catalytic activity is also influenced by the CTT [40].

Finally, using an in vitro sedimentation assay, the effect of C109 on the *S. aureus* FtsZ polymerization was characterized. Formerly, it was demonstrated that in *B. cenocepacia*, the compound is effective in inhibiting the polymerization of the purified FtsZ protein, preventing the formation of the Z-ring [24]. In *S. aureus*, C109 does not influence the in vitro polymerization of the purified protein, highlighting a significant difference in the effect of the compound on the two proteins. Considering the percentage of identity of the two proteins (46%) and the significant difference in their N- and C-terminal parts, it is possible to hypothesize that C109 interacts in a different way with the two homologous proteins. However, structural studies will be necessary to demonstrate this hypothesis. Moreover, the sedimentation assay showed that the truncated FtsZ of *S. aureus* is not able to polymerize in the tested conditions, confirming previously described data in which this version of the protein was able to form polymers very slowly and only after a long lag time [42,43].

In conclusion, the FtsZ inhibitor C109 demonstrated very good activity against the Gram-positive *S. aureus*, proving effective against clinical isolates and multiresistant strains. Moreover, its promising antibiofilm activity in a wound model of infection makes the development of a formulation for topical application worthy of further investigation. On the other hand, structural studies would greatly contribute to confirming the molecular activity of the compound on its target and designing derivatives with improved chemical properties.

## 4. Materials and Methods

### 4.1. Bacterial Strains and Culture Media

*Staphylococcus aureus* ATCC 25923 (MSSA) and Mu50 (MRSA with reduced susceptibility to vancomycin) [44] strains were used. Bacteria were grown aerobically in tryptic soy broth (TSB, BD) medium at 37 °C and 200 rpm or maintained on tryptic soy agar (TSA, BD) plates. For biofilm formation experiments, bacteria were grown in TSB supplemented with 1% glucose. *Escherichia coli* BL21(DE3) strain (laboratory collection) was used for recombinant protein overexpression and grown using Luria–Bertani (LB, BD) broth at 37 °C with shaking or on LB agar plates. The antibiotic kanamycin (PanReac, AppliChem, ITW Reagents, Glenview, IL, USA) was used at 50 μg/mL for plasmid selection and maintenance in *E. coli*.

### 4.2. Minimum Inhibitory Concentration (MIC) and Minimum Bactericidal Concentration (MBC) Determination for S. aureus Clinical Isolates

Minimum inhibitory concentrations (MICs) were determined in quadruplicates for a total of 102 *S. aureus* isolates (*n* = 51, 50% MSSA; *n* = 51, 50% MRSA) and *S. aureus* ATCC 25923 using broth microdilution method according to EUCAST guidelines, in Muller–Hinton (MH, Difco, BD, Franklin Lakes, NJ, USA) broth [45]. C109 was dissolved in pure DMSO (≥99.9%). Two-fold serial dilutions of C109 in concentrations ranging from 64  to 0.125 µg/mL with a final inoculum of 5 × 10^5^ CFU/mL were dispensed in each well of the 96-well culture plate. After incubation for 24 h at 35 °C, 30 µL of 0.015% resazurin (Merck, Readington, NJ, USA) were added to all wells, and the culture was further incubated for 2–4 h for the observation of blue to pink color change, indicating bacterial growth [46]. MICs were determined from visual reading before and after adding resazurin as the lowest concentration able to inhibit microbial growth. All experiments were performed by two workers in quadruplicate, and the *S. aureus* ATCC 25923 strain was included in each plate.

Viable colony counts were performed in duplicates for each strain by diluting 2.5 µL from the growth-control well after inoculation in 50 µL of sterile distilled water and spreading on MH agar. Plate count was made by counting the colony-forming units (CFUs) after incubation for 24 h at 35 °C.

The minimum bactericidal concentrations (MBCs) of C109 were determined by subculturing 10 µL of bacterial culture from wells corresponding to the MIC value and two concentrations higher than the MIC value on MH agar plates. After 24 h of incubation in aerobic conditions at 35 °C, the lowest concentration of C109 that yielded no visible bacterial growth on MH agar plates was recorded as the MBC value.

### 4.3. In Vitro Biofilm Inhibition Test in 96-Well Microtiter Plates

The biofilm inhibitory activity of C109 compound was tested on *S. aureus* 25923 strain using the crystal violet staining method [47]. The bacterial cells were cultured in TSB + 1% glucose O/N at 37 °C and diluted to 10^7^–10^8^ CFU/mL; then, 100 μL of culture was pipetted into the microtiter plate either in the absence or presence of different concentrations of C109 (0.0306, 0.0612, 0.125, 0.250, 0.5, 1, 2, and 4 μg/mL). After 2 h of incubation, the supernatant (containing nonadherent cells) was removed and 100 μL of fresh sterile medium (containing the same concentration of C109) was added to each well and incubated for an additional 20 h at 37 °C. Biofilm biomass was quantified by staining with crystal violet and absorbance measurements at OD_600nm_. Results were expressed as the ratio between biofilm absorbance and planktonic bacteria absorbance normalized on the value obtained for cells treated with the vehicle (DMSO).

### 4.4. Biofilm Eradication Assay in 96-Well Microtiter Plates

To evaluate the biofilm eradication potential of C109 on *S. aureus* 25923, the protocol described in [25] was used with minor modifications. Bacteria were cultured O/N in TSB + 1% glucose and diluted to an OD_600_ = 0.05 (about 1 × 10^7^ CFU/mL) in the same medium. Then, 100 μL of bacterial suspension was added to the wells of a round-bottom 96-well microtiter plate and incubated for 1 h at 37 °C, allowing the bacterial adhesion to the abiotic surface. After that, wells were emptied, gently rinsed with 100 μL of physiological solution (PS), and filled with 100 μL of fresh medium before incubating the microplate for an additional 23 h at 37 °C. The day after, the supernatants were removed, and the biofilms were washed three times with 100 μL of PS. TSB medium containing 16 and 32 μg/mL of C109 was added to the mature biofilms, and the plate was incubated for 24 h at 37 °C. Each condition was tested in triplicate, and untreated controls (TSB alone) or antibiotic controls (TSB with 64 μg/mL of vancomycin or linezolid) were prepared. After 24 h, wells were rinsed three times with PS, completely removing planktonic bacteria, and the biofilms were disrupted by two cycles of vortexing (800 rpm, 5 min) and sonication (5 min, Bransonic 220; Branson Ultrasonics Corp., Danbury, CT, USA). The resulting microbial suspensions were serially diluted and plated onto LB agar for the determination of the CFU/mL.

### 4.5. Biofilm Evaluation by Confocal Laser Scanning Microscopy

Bacteria were cultured O/N in TSB + 1% glucose and diluted to an OD_600_ = 0.05 (about 1 × 10^7^ CFU/mL) in the same medium. Bacterial suspension was added to the four-well Nunc Lab-Tek II Chambered Coverglass for two hours in TSB + 1% glucose, at 37 °C, in presence of different concentrations of C109 (0.625 or 1 μg/mL). The medium was removed, the biofilm was washed once in PBS to remove nonadherent cells, and fresh TSBG medium containing the same concentration of C109 was added. After an overnight incubation, the medium was removed, and biofilms were washed twice with PBS and stained with Syto 9 (Invitrogen) at a final concentration of 5 μM. A 63× oil immersion objective and a Leica DMi8 with 500- to 530-nm (green fluorescence representing Syto 9) emission filters were used to take five snapshots randomly at different positions in the confocal field of each chamber. The Z-slices were obtained every 0.3 microns. For visualization and processing of biofilm images, ImageJ was used. The thickness, biomass, roughness coefficient, and biofilm distribution were measured using the COMSTAT 2 software [48]. All confocal scanning laser microscopy experiments were performed three times, and standard deviations were measured.

### 4.6. Preparation of the Artificial Dermis for the Biofilm Wound Model

The artificial dermis (AD) spongy sheets for the chronic wound biofilm model were prepared as previously described [30]. The AD is composed of hyaluronic acid (HA, Lifecore Biomedical, Chaska, MN, USA) and collagen type I (Merck, Readington, NJ, USA) and is divided into two layers. The upper one consists of a spongy sheet of high-molecular-weight HA (HMW-HA), obtained by chemical cross-linking of the HA molecules using ethylene glycol diglycidyl ether (EX810, Merck, Readington, NJ, USA). The lower one is composed of a mixture of HMW-HA, low-molecular-weight HA, and heat-denatured collagen. The two-layered AD sheets were prepared following several steps of freeze-drying, and subsequently, they were exposed on both sides to UV light to cross-link the collagen molecules and obtain the final sponges of approximately 1 cm^3^. Finally, the AD sheets were sterilized at 110 °C for 1 h [30].

### 4.7. S. aureus Mu50 Biofilm Inhibition in a Wound Model

The biofilm inhibitory activity of the C109 compound was tested against *S. aureus* Mu50 in an artificial wound infection model [30,48]. The AD, used as a substrate for the biofilm formation, was placed into flat-bottom 24-well plates, and 500 μL of chronic wound medium (Bolton broth (LabM) medium containing 50% bovine plasma (Merck, Readington, NJ, USA) and 5% freeze-thaw laked horse blood (Biotrading, Mijdrecht, Netherlands) stabilized with heparin (Merck, Readington, NJ, USA) was added dropwise on the top of it, allowing the complete adsorption of the liquid. Then, 10 μL of a bacterial O/N culture diluted to 1 × 10^6^ CFU/mL in PS was spotted on the upper surface of the AD, and 100 μL of a C109 solution with a concentration of 80 μg/mL (to obtain a final concentration of 8 μg/mL in the total well volume of 1 mL) was added. In order to prevent the dehydration of the AD sponges during the incubation, an additional 400 μL of the above-described chronic wound medium was added around it. After that, the plate was incubated at 37 °C for 24 h to allow the biofilm formation in a chronic wound-like environment. Growth controls (no treatment) and solvent-only controls (DMSO 0.8% in PS) were included, and each condition was prepared in triplicate. After one day of incubation, AD was rinsed once with PS to remove planktonic bacteria and subsequently transferred into a tube containing 10 mL of PS. Sessile cells were detached from the AD by three cycles of vortexing (30 s) and sonication (30 s; Branson 3510; Branson Ultrasonics Corp., Danbury, CT, USA), serially diluted, and plated on TSA to quantify the CFU/biofilm.

### 4.8. Cloning, Expression, and Purification of the S. aureus 25923 FtsZ Proteins

The full-length (1173 bp) and the truncated (912 bp) versions of the *ftsZ* gene of *S. aureus* 25923 were amplified from the genomic DNA by PCR using the primers SASUMOfor (5′-GAACAGATTGGTGGTATGTTAGAATTTGAA-3′) and SASUMOrev (5′-TACCTAAGCTTGTCTTTAACGTCTTGTTCT-3′) or SASUMOFtsZTRfor (5′-GAACAGATTGGTGGTTTAGCGACTTTAAAG-3′) and SASUMOFtsZTRrev (5′-TACCTAAGCTTGTCTTTAATCAAAACCAGTTGC-3′). These were designed following the In-fusion HD Cloning Kit protocol instructions (Takara Bio Inc., Shiga, Japan). The obtained fragments were then cloned separately into linearized pETSUMO vectors (Invitrogen) by recombination, according to the In-fusion HD Cloning Kit protocol (Takara Bio Inc., Shiga, Japan). The *E. coli* BL21(DE3) strain was used for the expression of the two recombinant proteins. After transformation, bacteria were grown O/N, inoculated 1/50 in 4.5 L of LB supplemented with kanamycin (50 μg/mL), and incubated at 37 °C with shaking until OD_600_ = 0.6 was reached. Then, the heterologous protein expression was induced with 1 mM of isopropyl β-D-thiogalactopyranoside (IPTG, Fisher Scientific, Waltham, Massachusetts), and the culture was grown O/N at 25 °C. Cells were harvested by centrifugation, resuspended in lysis buffer (50 mM Tris-HCl pH = 8, 300 mM KCl, 5 mM imidazole, and 10% glycerol) supplemented with 1 mM of the nonspecific protease inhibitor phenylmethanesulfonyl fluoride (PMSF, Merck, Readington, NJ, USA), and lysed by sonication. The lysate was clarified by centrifugation at 50,000× *g* for 40 min, and the supernatant was loaded on a HisTrap HP nickel column (1 mL, GE Healthcare, Chicago, IL, USA). The purified FtsZ protein was eluted with the lysis buffer containing 250 mM imidazole and subsequently dialyzed O/N against the lysis buffer supplemented with 1 mM dithiothreitol (DTT, PanReac ITW Reagents, Glenview, IL, USA) without imidazole. The SUMO protease was added to the dialysis to remove the SUMO tag. The protein was further purified by size exclusion chromatography using a HiLoad 16/60 Superdex-75 column (GE Healthcare, Chicago, IL, USA) in 20 mM Tris-HCl pH = 7.9, 50 mM KCl, 1 mM EDTA, 2.5 mM Mg(CH_3_COO)_2_, and 10% glycerol; quantified by Bradford protein assay; concentrated to 4 mg/mL; and stored at −80 °C until needed. The same protocol was used for both proteins.

### 4.9. In Vitro FtsZ GTPase Activity

GTPase activity was assayed at 30 °C using a pyruvate kinase–L-lactic dehydrogenase (PK/LDH) spectrophotometric coupled assay, as previously described [24] with minor modification. The reaction mixture was first set up to contain 50 mM MES (pH 6.5), 5 mM Mg(CH_3_COO)_2_, 100 mM CH_3_CO_2_K, 10 U PK/LDH, 0.25 mM NADH, 0.25 mM phosphoenolpyruvate, and 4.8 μM of wild-type or deleted FtsZ. The assay was initiated by the addition of 1 mM GTP. The experiments were performed in triplicate, and the kinetic constants were determined by fitting the data to the Michaelis–Menten equation using Prism 9. C109 was added in concentrations ranging from 0.5 to 100 μM, and the inhibitory concentration that reduced the enzymatic activity by half (IC50) was determined using Prism 9.

### 4.10. In Vitro FtsZ Polymerization Assay

The polymerization of wild-type and deleted FtsZ was assessed in vitro using a sedimentation protocol, as previously described [24]. The reaction mixture was set up to contain 50 mM MES (pH 6.5), 5 mM Mg(CH_3_COO)_2_, 100 mM CH_3_CO_2_K, 12 μM SaFtsZ, and 2 mM GTP or GDP. The reaction mixtures were incubated for 10 min at 30 °C and 300 rpm to allow the polymerization to occur. Subsequently, samples were ultracentrifuged at 350,000× *g* for 10 min at 25 °C, and the supernatant was immediately separated from the pellet, which contained the protein polymers. The samples were analyzed by SDS-PAGE on 12% polyacrylamide gels. The in vitro polymerization of both versions of the FtsZ protein was tested in the presence of 50 μM of C109.

## Data Availability

Data supporting the conclusions of this article are included within the article and the additional files.

## Supplementary Materials

The following are available online at https://www.mdpi.com/article/10.3390/pathogens10070886/s1, Table S1: Samples, wards, minimum inhibitory concentrations (MICs), and minimum bactericidal concentrations (MBCs) of C109 against 51 MSSA clinical isolates, Table S2: Samples, wards, minimum inhibitory concentrations (MICs) and minimum bactericidal concentrations (MBCs) of C109 against 51 MRSA clinical isolates, Figure S1: Effect of C109 against *S. aureus* ATCC 25923 biofilm, Figure S2: Protein sequence alignment of *S. aureus* FtsZ (FtsZSa) and *B. cenocepacia* FtsZ (FtsZBc).
