# Supplementary material for: Antistaphylococcal Activity of the FtsZ Inhibitor C109
Source: Pathogens. 2021 Jul 13;10(7):886. doi: 10.3390/pathogens10070886 (PMC8308607; doi:10.3390/pathogens10070886)
Supplement: Supplementary file 1 [file pathogens-10-00886-s001.zip › pathogens-1285291-supplementary.pdf]

Supplementary data

Table S1. Samples, wards, Minimum Inhibitory Concentrations (MIC) and Minimum Bactericidal Concentrations (MBC) of C109 for 51 MSSA clinical isolates

S. aureus* strain	**Sample**	**Ward^**	**MIC (µg/ml)**	**MBC (µg/ml)**
**548A SM PV 08**	blood culture	Medicine	1	1
**548 SM PV 08**	blood culture	Medicine	2	2
**554 SM PV 08**	blood culture	Medicine	2	2
**554A SM PV 08**	blood culture	Medicine	2	2
**629 SM PV 08**	blood culture	Hematology	2	4
**556 SM PV 08**	blood culture	Pneumology	4	4
**557 SM PV 08**	blood culture	Medicine	4	4
**10 AM LC 10**	blood culture	ICU	4	4
**563 SM PV 08**	blood culture	Surgery	8	8
**561 SM PV 08**	blood culture	Pneumology	8	8
**31 AM LC 10**	blood culture	Rehabilitation	8	16
**16 AM LC 10**	bronchial aspirate	ICU	2	2
**15 AM LC 10**	bronchial aspirate	ICU	2	2
**14 AM LC 10**	bronchial aspirate	ICU	8	8
**599 SM PV 08**	skin swab	Neonatal Pathology	1	2
**579 SM PV 08**	skin swab	Hematology	2	2
**628 SM PV 08**	skin swab	External ambulatory	2	2
**379 SM PV 07**	skin swab	External ambulatory	4	4
**579A SM PV 08**	skin swab	Hematology	4	4
**598A SM PV 08**	skin swab	Medicine	4	8
**600 SM PV 08**	skin swab	Medicine	4	8
**22 AM LC 10**	skin swab	Medicine	8	8
**577 SM PV 08**	sputum	Medicine	2	2
**577A SM PV 08**	sputum	Medicine	2	2
**550 SM PV 08**	sputum	Medicine	4	8
**590 SM PV 08**	ulcer swab	External ambulatory	2	2
**355 SM PV 07**	ulcer swab	External ambulatory	4	8
**549 SM PV 08**	ulcer swab	Hematology	4	4
**551 SM PV 08**	ulcer swab	Medicine	4	4
**558 SM PV 08**	ulcer swab	External ambulatory	4	4
**598 SM PV 08**	ulcer swab	Medicine	4	4
**26 AM LC 10**	ulcer swab	LTCF	4	8
**13 AM LC 10**	urine	Nephrology	8	8
**623 SM PV 08**	vaginal swab	Obstetrics	4	4
**337 SM PV 07**	wound swab	Medicine	2	4
**27 AM LC 10**	wound swab	Orthopedic	2	2
**343 SM PV 07**	wound swab	-	4	>32
**339 SM PV 07**	wound swab	Vascular surgery	4	4

**363 SM PV 07**	wound swab	External ambulatory	4	4
**553 SM PV 08**	wound swab	External ambulatory	4	4
**560 SM PV 08**	wound swab	Ambulatory	4	4
**559 SM PV 08**	wound swab	Medicine	4	4
**580A SM PV 08**	wound swab	External ambulatory	4	4
**587 SM PV 08**	wound swab	Surgery	4	4
**606 SM PV 08**	wound swab	Dermatology	4	8
**351 SM PV 07**	wound swab	External ambulatory	8	>32
**366 SM PV 07**	wound swab	External ambulatory	8	8
**397 SM PV 07**	wound swab	External ambulatory	8	16
**580 SM PV 08**	wound swab	External ambulatory	8	32
**12 AM LC 10**	wound swab	Orthopedics	8	8
**402 SM PV 07**	-	Emergency	2	2

-: data not available
*: SM PV, Fondazione IRCCS Policlinico San Matteo, Pavia; AM LC, Alessandro Manzoni Hospital, Lecco; 07, 08, 10, year of strain's isolation
^: ICU, Intensive Care Unit; LTCF, Long Term Care Facility

Table S2. Samples, wards, Minimum Inhibitory Concentrations (MIC) and Minimum Bactericidal Concentrations (MBC) of C109 for 51 MRSA clinical isolates

S. aureus* strain	**Sample**	**Ward^**	**MIC (µg/ml)**	**MBC (µg/ml)**
**566 SM PV 08**	biological material	External ambulatory	1	1
**290 SM PV 06**	biological material	Medicine	4	4
**279 SM PV 06**	biological material	External ambulatory	8	8
**567 SM PV 08**	blood culture	Medicine	1	1
**11 AM LC 10**	blood culture	ICU	1	2
**316 SM PV 07**	blood culture	Oncology	2	4
**321 SM PV 07**	blood culture	-	2	2
**589 SM PV 08**	blood culture	Ematologia degenza	2	2
**644 SM PV 09**	blood culture	Medicine	2	4
**25 AM LC 10**	blood culture	Neurology	2	2
**265 SM PV 06**	blood culture	Hematology	4	8
**267 SM PV 06**	blood culture	Medicine	4	4
**320 SM PV 07**	blood culture	Surgery	4	4
**17 AM LC 10**	blood culture	Urology	4	8
**301 SM PV 06**	blood culture	Medicine	8	8
**9 AM LC 10**	blood culture	Medicine	8	8
**23 AM LC 10**	blood culture	Medicine	8	16
**30 AM LC 10**	bronchial aspirate	ICU	1	2
**1 AM LC 10**	bronchial aspirate	Medicine	4	16
**564 SM PV 08**	conjunctival swab	Neonatology	2	2

**315 SM PV 07**	drainages	Surgery	2	2
**326 SM PV 07**	drainages	Surgery	8	>32
**583 SM PV 08**	ear swab	External ambulatory	2	2
**334 SM PV 07**	pus	Urology	4	4
**286 SM PV 06**	pus	Surgery	8	8
**576 SM PV 08**	skin swab	Medicine	2	2
**7 AM LC 10**	skin swab	External ambulatory	2	8
**568 SM PV 08**	skin swab	Dermatology	4	16
**627 SM PV 08**	skin swab	Neonatal pathology	4	8
**284 SM PV 06**	skin swab	Dermatology	8	16
**570 SM PV 08**	sputum	Pneumology	1	2
**288 SM PV 06**	sputum	Medicine	2	2
**424 SM PV 08**	sputum	Medicine	4	4
**287 SM PV 06**	sputum	Ematology	8	16
**291 SM PV 06**	sputum	Medicine	8	16
**569 SM PV 08**	ulcer swab	External ambulatory	1	1
**19 AM LC 10**	ulcer swab	Medicine	1	1
**312 SM PV 07**	ulcer swab	External ambulatory	8	8
**341 SM PV 07**	urine	-	1	2
**565 SM PV 08**	urine	Medicine	1	1
**311 SM PV 07**	wound swab	Surgery	1	2
**562 SM PV 08**	wound swab	Pediatric surgery	1	1
**276 SM PV 06**	wound swab	Surgery	2	4
**303 SM PV 07**	wound swab	Vascular surgery	2	2
**306 SM PV 07**	wound swab	Surgery	2	2
**310 SM PV 07**	wound swab	Medicine	2	2
**356 SM PV 07**	wound swab	Orthopedics	2	4
**626 SM PV 08**	wound swab	Surgery	4	4
**633 SM PV 08**	wound swab	Ortopedic	4	4
**296 SM PV 06**	wound swab	Ematology	8	8
**24 AM LC 10**	wound swab	External ambulatory	8	8

-: data not available
*: SM PV, Fondazione IRCCS Policlinico San Matteo, Pavia; AM LC, Alessandro Manzoni Hospital, Lecco; 06, 07, 08, 09, 10, year of strain’s isolation
^: ICU, Intensive Care Unit

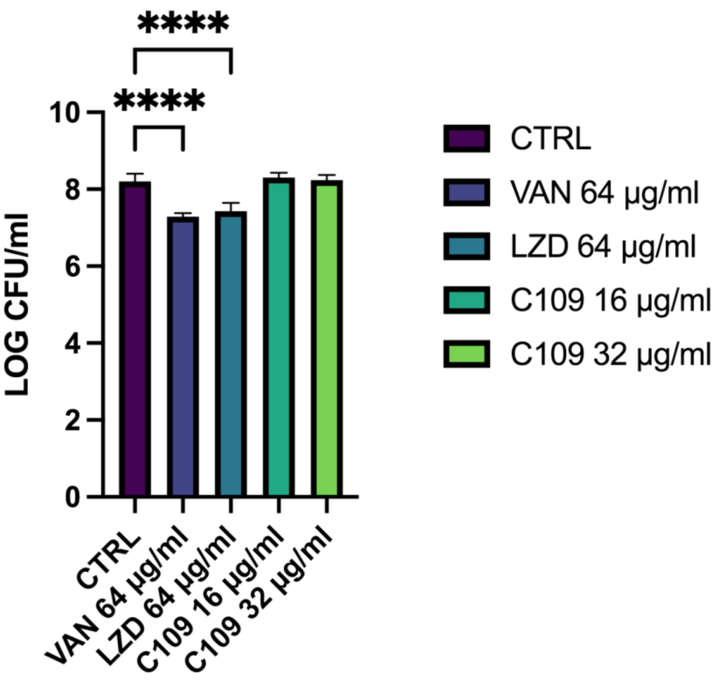

Figure S1. Effect of C109 against *S. aureus* ATCC 25923 biofilm eradication. Bacterial biofilms were treated with the compounds indicated below each bar. CTRL, no drug; VAN, vancomycin; LZD, linezolid. The results are expressed as LOG CFU/mL (mean ± standard error, n = 4). **** $p < 0.0001$ (one-way ANOVA test).

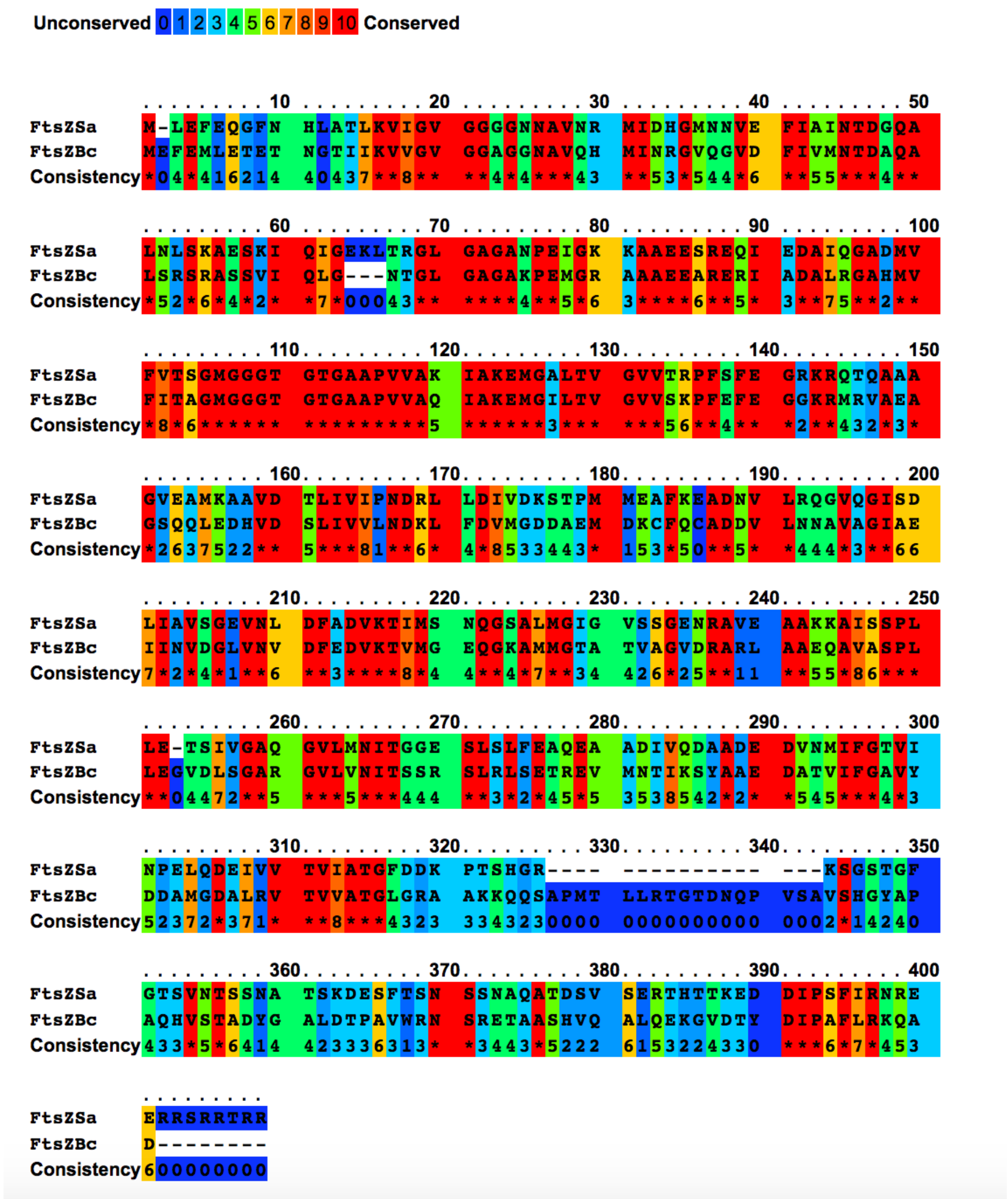

Figure S2. Protein sequence alignment of *S. aureus* FtsZ (FtsZSa) and *B. cenocepacia* FtsZ (FtsZBc). Less conserved residues are in blue while most conserved are in red. The alignment was obtained with PRALINE multiple sequence alignment [1].

1. Simossis, V.A.; Heringa, J. PRALINE: A multiple sequence alignment toolbox that integrates homology-extended and secondary structure information. *Nucleic Acids Res.* **2005**, *33*, W289–W294.
